# Risk and Population Attributable Fraction of Stroke Subtypes in Japan

**DOI:** 10.2188/jea.JE20220364

**Published:** 2024-05-05

**Authors:** Hiroshi Yatsuya, Kazumasa Yamagishi, Yuanying Li, Isao Saito, Yoshihiro Kokubo, Isao Muraki, Manami Inoue, Shoichiro Tsugane, Hiroyasu Iso, Norie Sawada

**Affiliations:** 1 Department of Public Health and Health Systems, Nagoya University Graduate School of Medicine, Nagoya, Japan; 2 Department of Public Health Medicine, Faculty of Medicine, and Health Services Research and Development Center, University of Tsukuba, Ibaraki, Japan; 3 Department of Public Health and Epidemiology, Faculty of Medicine, Oita University, Oita, Japan; 4 Department of Preventive Cardiology, National Cerebral and Cardiovascular Center, Osaka, Japan; 5 Public Health, Department of Social and Environmental Medicine, Graduate School of Medicine, Osaka University, Osaka, Japan; 6 Division of Prevention, National Cancer Center Institute for Cancer Control, Tokyo, Japan; 7 Division of Cohort Research, National Cancer Center Institute for Cancer Control, Tokyo, Japan; 8 International University of Health and Welfare Graduate School of Public Health, Tokyo, Japan; 9 The Institute for Global Health Policy, National Center for Global Health and Medicine, Tokyo, Japan

**Keywords:** cohort study, stroke, risk factor, population attributable fraction

## Abstract

**Background:**

Associations of major risk factors for stroke with total and each type of stroke, as well as subtypes of ischemic stroke, and their population attributable fractions had not been examined comprehensively.

**Methods:**

Participants of the Japan Public Health Center-based prospective (JPHC) Study Cohort II without histories of cardiovascular disease and cancer (*n* = 14,797) were followed from 1993 through 2012. Associations of current smoking, hypertension, diabetes, overweight (body mass index ≥25 kg/m^2^), non-high-density lipoprotein cholesterol (non-HDLC) categories, low HDLC (<40 mg/dL), urine protein, and history of arrhythmia were examined in a mutually-adjusted Cox regression model that included age and sex. Population attributable fractions (PAFs) were estimated using the hazard ratios and the prevalence of risk factors among cases.

**Results:**

Subjects with hypertension were 1.63 to 1.84 times more likely to develop any type of stroke. Diabetes, low HDLC, current smoking, overweight, urine protein, and arrhythmia were associated with risk of overall and ischemic stroke. Hypertension and urine protein were associated with risk of intracerebral hemorrhage, while current smoking, hypertension, and low non-HDLC were associated with subarachnoid hemorrhage. Hypertension alone accounted for more than a quarter of stroke incidence, followed by current smoking and diabetes. High non-HDLC, current smoking, low HDLC, and overweight contributed mostly to large-artery occlusive stroke. Arrhythmia explained 13.2% of embolic stroke. Combined PAFs of all the modifiable risk factors for total, ischemic, and large-artery occlusive strokes were 36.7%, 44.5%, and 61.5%, respectively.

**Conclusion:**

Although there are differences according to subtypes, hypertension could be regarded as the most crucial target for preventing strokes in Japan.

## INTRODUCTION

Stroke constitutes a major global burden of disease, although its risk factors have been extensively studied.^1^^–^^6^ In Japan, stroke was a leading cause of death until 1985 and the second until 2018.^7^ In spite of the continuous decline in the mortality rate, it is the second leading cause of long-term care requiring disability following dementia.^8^ In particular, it is still the leading cause of disability in men, as well as more severe disability in both men and women. To inform more efficient and effective programs to prevent the incidence, risk factor characterization for each type of stroke and quantifying their population impact would continue to be useful, since they are reportedly different according to subtypes, and distribution of the risk factors vary by periods and countries.^6^^,^^9^

In the present study, we evaluated the associations of major risk factors of stroke with total and each type of stroke, as well as subtypes of ischemic stroke, using a large-scale population-based cohort study in Japan.

## METHODS

### Participants

The Japan Public Health Center-based prospective Study (JPHC Study) is a nationally representative study that was established in 1990 (Cohort I) and 1993–1994 (Cohort II) in 11 public health-center areas throughout Japan, initially enrolling 140,420 individuals.^10^ In the present study, we used Cohort II data since the history of arrythmia, which was considered as a surrogate for atrial fibrillation, was not obtained in Cohort I. Of 61,980 (female: 31,526, male: 30,454) participants aged 40–69 years, 17,328 (female: 11,416, male: 5,912) subjects have information on health check-ups and among them 16,476 (female: 10,848, male: 5,628) subjects also have information on self-reported questionnaire at baseline. We excluded subjects with self-reported histories of stroke (*n* = 100), coronary heart disease (CHD) (*n* = 168), or cancer (*n* = 393) including 13 individuals with two or three histories, with missing values for smoking habits (*n* = 171) or self-reported history of arrhythmia (*n* = 1,034) were excluded, leaving 14,658 (female: 9,728, male: 4,930) subjects for the analysis. The study protocol, including the informed consent procedure of the JPHC study, was approved by the Human Ethics Review Committees of the National Cancer Center (approval number: 2001-021 and 2015-085) and Nagoya University (approval number: 2021-0222).

### Follow up

Subjects were followed-up from the date of the baseline survey through 2012, when the JPHC Study terminated its follow-up of cardiovascular disease incidence. The person-years were calculated from baseline to the date of ascertainment of the incident stroke, the date of moving out of the study area, incident CHD or death, or the end of the follow-up, whichever came first.

Residence and survival were ascertained annually using residential registries maintained by each municipality. In Japan, residency and death registration are required by law, and these registries are believed to be complete. Information on the cause of death was obtained through the death certificate provided by the Ministry of Health, Labour and Welfare after the Ministry of Internal Affairs and Communications granted permission.

### Ascertainment of stroke and classification of stroke types

All the major hospitals capable of managing acute stroke care located in the sampling areas of the study were registered. The medical records were reviewed regularly by physicians in registered hospitals or public health centers blinded to the risk factor statuses, using the standard format of the registry. A systematic review of the death certificate was conducted to complete surveillance for the incidence of fatal stroke. The stroke diagnosis and the incidence date were assigned according to criteria adopted from the National Survey of Stroke criteria, which require a constellation of neurological deficits of sudden or rapid onset lasting at least 24 hours or until death.^9^^,^^11^ A definitive diagnosis for each type of stroke was established based on computed tomography scans, magnetic resonance imaging, or documented in the autopsy report. A stroke was classified into ischemic stroke when an acute infarction occurred without evidence for either intracerebral hemorrhage or subarachnoid hemorrhage. Ischemic stroke was further classified into lacunar, large-artery occlusive, and embolic stroke. Lacunar included the detected infarct in basal ganglia, brain stem, thalamus, internal capsule, or cerebral white matter areas; large-artery occlusive stroke cortical areas. Embolic stroke depended on clinical diagnosis with the presence of an embolus in the brain or medical record evidence of a possible source of emboli, such as moderate or more severe valvular heart disease, atrial fibrillation, or intracardiac thrombus. Undetermined stroke cases were included for the definition of total or ischemic strokes.

### Baseline measurements of risk factors of stroke

Smoking status, history of arrhythmia, and medication use for hypertension or hyperglycemia were obtained from a self-administered questionnaire. The following items were obtained at the baseline health check-up: height and weight were measured, and body mass index (BMI) was calculated as weight (kg) divided by the square of the height in meters (m^2^). Blood pressure (BP) was measured using a standard mercury sphygmomanometer applied to the right arm of the seated participant after a 5-min rest. Either fasting (*n* = 5,665, 39% of total samples) or non-fasting blood samples were collected. Serum total and high-density lipoprotein cholesterol (TC and HDLC) and glucose were measured in laboratories. The non-HDLC level was obtained by subtracting HDLC from TC. The precision and accuracy of lipid measurement in all laboratories were satisfactory, according to the Osaka Medical Center for Cancer and Cardiovascular Diseases, a member of the Cholesterol Reference Method Laboratory Network.^12^ Smoking was dichotomized into current-smoker and non-smokers (reference). BMI was categorized into <18.5, 18.5–<25 (reference), and ≥25 kg/m^2^. Diabetes was defined as a fasting glucose level ≥126 mg/dL or casual glucose level ≥200 mg/dL or medication use of hyperglycemia. Hypertension was defined as systolic blood pressure ≥140 mm Hg or diastolic blood pressure ≥90 mm Hg or taking antihypertensive medication. HDLC was dichotomized into <40 and ≥40 mg/dL (reference). Non-HDLC was categorized into four groups: <130, 130–149 (reference), 150–169, and ≥170 mg/dL. The non-HDLC ≥170 mg/dL category includes subjects who reported the use of lipid-lowering medication. Urinary protein was defined as dipstick proteinuria (trace or more). History of arrhythmia was self-reported. It was inquired by a question: “Have you ever been told about an irregularity of your heart beat?” Data on electrocardiographic findings were not obtained in the JPHC Study. Since it was considered that risks related to high normal and elevated blood pressure require elucidation, a supplementary analysis was conducted using blood pressure classification of the Japanese Society of Hypertension Guidelines for the Management of Hypertension (JSH 2019).^13^^,^^14^

### Statistical analysis

The area-stratified multivariable-adjusted Cox proportional hazards model was used to estimate the hazard ratio (HR) and 95% confidence interval (CI) as an indicator of relative risk (RR). All eight risk factors of interest as well as continuous age (40–69 years) and sex, were simultaneously included in the model to obtain adjusted RR for an overall and specific type of stroke. Sex-stratified analyses were carried out as supplementary analyses.

Population attributable fraction (PAF; %) was estimated to evaluate the possible impact of altering single risk factors on the overall and specific type of stroke in the study population. PAF was calculated using the Levin’s attributable risk formula: PAF = pd_i_[(RRa_i_ − 1)/RRa_i_], where pd_i_ is the prevalence of ith exposure level among cases, and RRa_i_ is the adjusted RR comparing ith exposure level with an unexposed group (i = 0). The 95% CI was computed using formulas proposed by Sander Greenland.^15^ Combined PAF of all the modifiable risk factors were calculated based on the method proposed by Dietrich Plass.^16^

In an attempt to quantify the current health burden of the selected risk factors (ie, hypertension, diabetes, current smoking, non-HDLC of 150–169 mg/dL or ≥170 mg/dL, BMI ≥25 kg/m^2^) based on the availability of representative prevalence data in the current Japanese population, PAFs2019 were calculated using the prevalence of risk factors (Pe_i_) reported in the National Health and Nutrition Survey in Japan 2019^17^ based on the following function: Pe_i_ (RRa_i_ − 1)/(Pe_i_ (RRa_i_ − 1) + 1). However, it should be noted that bias could exist in PAFs2019 calculated using adjusted HR.^18^

All analyses were conducted using SAS for Windows, version 9.4 (SAS Institute, Cary, NC, USA), with two-sided *P*-values <0.05 considered significant.

## RESULTS

The mean age of the subjects was 57.3 (standard deviation, 8.2) years and 33.6% were men. The most prevalent risk factors are non-HDLC ≥150 (ie, 150–169 or ≥170) mg/dL (45%) and hypertension (44%), followed by BMI ≥25 kg/m^2^ (32%), current smoking (15%), HDLC <40 mg/dL (10%), arrhythmia (8.2%), urine protein (7.1%), and diabetes mellitus (4.5%) (Table 1).

**Table 1.** Baseline characteristics of the study participants and the number and incidence rate of stroke subtypes, JPHC Study, 1993–1994

	*n* of subjects (%)	*n* of stroke cases (incidence rate (/100,000 person-years))						

		Total Stroke	Intracerebral hemorrhage	Subarachnoid hemorrhage	Ischemic stroke	Embolic stroke	Lacunar stroke	Large-artery occlusive stroke
Age 40–49 years	3,047 (20.8)	61 (107.8)	21 (37.1)	9 (15.9)	31 (54.8)	10 (17.7)	17 (30.0)	4 (7.1)
Age 50–59 years	4,618 (31.5)	195 (229.5)	69 (81.2)	16 (18.8)	106 (124.8)	30 (35.3)	41 (48.3)	29 (34.1)
Age 60–69 years	6,993 (47.7)	659 (561.5)	161 (137.2)	45 (38.3)	435 (370.6)	139 (118.4)	175 (149.1)	88 (75.0)
Women	9,728 (66.4)	468 (265.4)	117 (66.4)	58 (32.9)	285 (161.6)	93 (52.7)	119 (67.5)	55 (31.2)
Men	4,930 (33.6)	447 (541.1)	134 (162.2)	12 (14.5)	287 (347.4)	86 (104.1)	114 (138.0)	66 (79.9)
Non-smoking	12,395 (84.6)	717 (322.7)	210 (94.5)	60 (27.0)	432 (194.4)	145 (65.3)	179 (80.6)	83 (37.4)
Current smoking	2,263 (15.4)	198 (539.6)	41 (111.7)	10 (27.3)	140 (381.5)	34 (92.7)	54 (147.2)	38 (103.6)
Hypertension (−)	8,200 (55.9)	339 (229.5)	97 (65.7)	28 (19.0)	208 (140.8)	61 (41.3)	82 (55.5)	48 (32.5)
Hypertension (+)	6,458 (44.1)	576 (518.0)	154 (138.5)	42 (37.8)	364 (327.3)	118 (106.1)	151 (135.8)	73 (65.6)
Diabetes mellitus (−)	13,994 (95.5)	825 (332.4)	237 (95.5)	67 (27.0)	503 (202.7)	158 (63.7)	200 (80.6)	108 (43.5)
Diabetes mellitus (+)	664 (4.5)	90 (839.2)	14 (130.5)	3 (28.0)	69 (643.4)	21 (195.8)	33 (307.7)	13 (121.2)
Non-HDLC <130 mg/dL	3,227 (22.0)	191 (33.4)	53 (9.3)	22 (3.8)	112 (19.6)	55 (9.6)	35 (6.1)	14 (2.4)
Non-HDLC 130–149 mg/dL	4,892 (33.4)	306 (35.8)	95 (11.1)	25 (2.9)	178 (20.8)	68 (8.0)	61 (7.1)	33 (3.9)
Non-HDLC 150–169 mg/dL	2,746 (18.7)	158 (32.3)	45 (9.2)	7 (1.4)	102 (20.9)	36 (7.4)	30 (6.1)	31 (6.3)
Non-HDLC ≥170	3,793 (25.9)	260 (38.5)	58 (8.6)	16 (2.4)	180 (26.7)	74 (11.0)	53 (7.9)	43 (6.4)
HDLC ≥40 mg/dL	13,255 (90.4)	792 (336.8)	222 (94.4)	62 (26.4)	489 (208.0)	150 (63.8)	207 (88.0)	96 (40.8)
HDLC <40 mg/dL	1,403 (9.6)	123 (517.1)	29 (121.9)	8 (33.6)	83 (348.9)	29 (121.9)	26 (109.3)	25 (105.1)
Urine protein (−)	13,622 (92.9)	812 (336.2)	220 (91.1)	64 (26.5)	508 (210.4)	159 (65.8)	210 (87.0)	105 (43.5)
Urine protein (+)	1,036 (7.1)	103 (591.3)	31 (178.0)	6 (34.4)	64 (367.4)	20 (114.8)	23 (132.0)	16 (91.9)
Arrhythmia (−)	13,456 (91.8)	812 (340.5)	232 (97.3)	62 (26.0)	498 (208.9)	141 (59.1)	212 (88.9)	111 (46.6)
Arrhythmia (+)	1,202 (8.2)	103 (503.1)	19 (92.8)	8 (39.1)	74 (361.4)	38 (185.6)	21 (102.6)	10 (48.8)
BMI <18.5 kg/m^2^	512 (3.5)	25 (293.2)	7 (82.1)	2 (23.5)	15 (175.9)	8 (93.8)	6 (70.4)	1 (11.7)
BMI, 18.5–<25 kg/m^2^	9,414 (64.2)	553 (333.5)	161 (97.1)	39 (23.5)	342 (206.3)	104 (62.7)	146 (88.1)	63 (38.0)
BMI ≥25 kg/m^2^	4,732 (32.3)	337 (398.4)	83 (98.1)	29 (34.3)	215 (254.2)	67 (79.2)	81 (95.8)	57 (67.4)

BMI, body mass index; HDLC, high-density lipoprotein cholesterol; JPHC Study indicates Japan Public Health Center-based Prospective Study.

Hypertension was defined as systolic blood pressure ≥140 mm Hg, diastolic blood pressure ≥90 mm Hg, or the use of antihypertensive medication. Diabetes mellitus was defined as fasting blood glucose ≥126 mg/dL, casual blood glucose ≥200 mg/dL, or the use of antidibetic medication. The non-HDLC ≥170 mg/dL category includes subjects who reported the use of lipid-lowering medication.

During 258,914 person-years of follow-up (median, 20 years), a total of 915 incident strokes (male: 447, female: 468) were recorded, including 572 ischemic (62.5%), 251 intracerebral hemorrhagic (27.4%), and 70 subarachnoid hemorrhagic strokes (7.7%). Ischemic strokes constituted 233 lacunars (40.7% of ischemic strokes), 179 embolic (31.3%), and 121 large-artery occlusive strokes (21.2%) (Table 1).

Table 2 presents mutually-adjusted HRs and PAFs for total stroke and stroke subtypes. Subjects with hypertension were 1.63 to 1.84 times more likely to develop any stroke. Diabetes mellitus was associated with 1.83 to 2.25 times higher risk of developing total and ischemic stroke. Also, HDLC <40 mg/dL, current smoking, urine protein (marginal significance), and arrhythmia were associated with total and ischemic stroke, with HRs ranging from 1.27 to 1.50. In addition, those with BMI ≥25 kg/m^2^ had 1.16 and 1.22 times higher risk of developing total and ischemic stroke than those without, respectively. In contrast, current smoking was associated with an increased risk of subarachnoid hemorrhage (HR 2.32; 95% CI, 1.02–5.26). Non-HDLC ≥170 mg/dL was associated with increased risk of ischemic stroke (HR 1.27; 95% CI, 1.00–1.62). In contrast, non-HDLC categories ≥150 mg/dL were associated with decreased risk of subarachnoid hemorrhage compared to 130–149 mg/dL group. Sex-stratified analysis indicated that inverse association between non-HDLC and subarachnoid hemorrhage existed only in women (*P* for interaction by sex = 0.77). Although the associations were generally similar between men and women, hypertenson in men and current smoking in women were more strongly associated with risk of subarachnoid hemorrhage (eTable 1A and eTable 1B). Also, urine protein’s association with risk of ischemic stroke was weaker in men compared to that in women.

**Table 2.** Multivariable-adjusted hazard ratios and population attributable fractions for stroke subtypes, JPHC Study, 1993–2012

Risk factors	Total Stroke		Ischemic stroke		Intracerebral hemorrhage		Subarachnoid hemorrhage	
	*n* of cases = 915		*n* of cases = 572		*n* of cases = 251		*n* of cases = 70	

	HR (95% CI)	PAF (95% CI)	HR (95% CI)	PAF (95% CI)	HR (95% CI)	PAF (95% CI)	HR (95% CI)	PAF (95% CI)
Men vs women	1.58 (1.36–1.84)		1.63 (1.34–1.98)		2.08 (1.58–2.74)		0.26 (0.12–0.55)	
Age per 1 year	1.09 (1.08–1.10)		1.11 (1.09–1.12)		1.06 (1.04–1.08)		1.05 (1.02–1.09)	
Current smoking vs non-smoking	1.33 (1.11–1.60)	5.4 (1.8–8.9)	1.56 (1.25–1.95)	8.8 (4.0–13.3)	0.83 (0.57–1.20)	−3.4 (−10.0 to 2.8)	2.32 (1.02–5.26)	8.1 (−1.6 to 16.9)
Hypertension	1.69 (1.47–1.95)	25.8 (19.0–31.9)	1.63 (1.36–1.94)	24.5 (15.6–32.5)	1.84 (1.41–2.39)	27.9 (15.4–38.6)	1.75 (1.05–2.90)	25.7 (−0.1 to 44.8)
Diabetes mellitus	1.83 (1.47–2.29)	4.5 (2.4–6.5)	2.25 (1.74–2.91)	6.7 (3.8–9.5)	1.03 (0.60–1.78)	0.2 (−2.9 to 3.2)	0.90 (0.28–2.89)	−0.5 (−5.7 to 4.4)
Non-HDLC <130 vs 130–149 mg/dL	1.17 (0.97–1.40)	4.7 (−1.0, 10.2)	1.14 (0.90–1.45)	3.8 (−3.3 to 10.4)	1.30 (0.93–1.83)	8.7 (−2.9 to 19.1)	0.94 (0.53–1.69)	−2.2 (−26.0 to 17.2)
Non-HDLC 150–169 vs 130–149 mg/dL	0.96 (0.77–1.18)	−0.8 (−4.6, 2.8)	1.06 (0.81–1.38)	0.9 (−3.8 to 5.5)	1.01 (0.68–1.51)	0.2 (−7.2 to 7.1)	0.34 (0.14–0.79)	−19.9 (−34.5 to −6.8)
Non-HDLC ≥170 vs 130–149 mg/dL	1.08 (0.89–1.31)	2.1 (−3.2, 7.1)	1.27 (1.00–1.62)	6.7 (0.0–13.1)	0.93 (0.64–1.35)	−1.8 (−11.3 to 6.9)	0.49 (0.26–0.94)	−23.7 (−48.0 to −3.4)
HDLC <40 vs ≥40 mg/dL	1.27 (1.05–1.55)	2.9 (0.3–5.4)	1.28 (1.01–1.63)	3.2 (−0.2 to 6.4)	1.27 (0.86–1.89)	2.5 (−2.1 to 6.8)	1.30 (0.61–2.77)	2.7 (−6.0 to 10.6)
Urine protein (+)	1.32 (1.07–1.64)	2.9 (0.4–5.0)	1.29 (0.99–1.69)	2.5 (−0.4 to 5.4)	1.47 (1.00–2.17)	4.0 (−0.7 to 8.4)	1.30 (0.55–3.07)	2.0 (−5.5 to 8.9)
Arrhythmia	1.33 (1.08–1.64)	2.8 (0.5–5.1)	1.50 (1.17–1.92)	4.3 (1.2–7.3)	0.96 (0.60–1.53)	−0.3 (−4.0 to 3.2)	1.37 (0.65–2.88)	3.1 (−5.5 to 10.9)
BMI <18.5 vs 18.5–<25 kg/m^2^	0.83 (0.56–1.24)	−0.6 (−1.7 to 0.6)	0.80 (0.47–1.34)	−0.7 (−2.1 to 0.7)	0.88 (0.41–1.88)	−0.4 (−2.6 to 1.7)	0.83 (0.20–3.45)	−0.6 (−4.8 to 3.5)
BMI ≥25 vs 18.5–<25 kg/m^2^	1.16 (1.00–1.33)	4.9 (−0.1 to 9.8)	1.22 (1.02–1.46)	6.8 (0.3–12.8)	0.90 (0.68–1.19)	−3.9 (−14.1 to 5.4)	1.44 (0.87–2.38)	12.6 (−7.2 to 28.7)

BMI, body mass index; CI, confidence interval; HDLC, high-density lipoprotein cholesterol; HR, hazard ratio; JPHC Study, Japan Public Health Center-based Prospective Study; PAF, population attributable fraction; vs, versus.

Cox proportional hazard model that was mutually adjusted for all risk factors was used.

Hypertension was defined as systolic blood pressure ≥140 mm Hg, diastolic blood pressure ≥90 mm Hg, or the use of antihypertensive medication. Diabetes mellitus was defined as fasting blood glucose ≥126 mg/dL, casual blood glucose ≥200 mg/dL or the use of antidiabetic medication. The non-HDLC ≥170 mg/dL category includes subjects who reported the use of lipid-lowering medication.

PAF estimates indicated that hypertension would be the single most important risk factor for the incidence of total stroke and its subtypes. Its impact seemed consistent across all stroke subtypes by explaining approximately a quarter of stroke cases. Current smoking and diabetes mellitus represented the second and third largest PAFs for total stroke (5.4% and 4.5%, respectively). Similarly, current smoking, BMI ≥25 kg/m^2^, and diabetes mellitus accounted for 8.8%, 6.8%, and 6.7% of ischemic stroke, respectively. Arrhythmia explained 2.8% of total stroke and 4.3% of ischemic stroke. Both urine protein and HDLC <40 mg/dL explained 2.9% of total stroke. The PAFs2019 of hypertension for total stroke was about 3 percentage points lower, whereas diabetes mellitus was 4 percentage points higher than those estimated using risk factor prevalence in the present study (eTable 2).

Table 3 shows mutually-adjusted HRs and PAFs for ischemic stroke subtypes. Diabetes mellitus was related to all subtypes of ischemic stroke with the HRs of 2.68 (95% CI, 1.84–3.91) for lacunar, 2.17 (95% CI, 1.36–3.47) for embolic, and 2.00 (95% CI, 1.11–3.61) for large-artery occlusive stroke. Hypertension was associated with 1.77 (95% CI, 1.34–2.34) and 1.76 (95% CI, 1.27–2.42) times higher risk of lacunar and embolic stroke, respectively. Additionally, subjects with HDLC <40 mg/dL or arrhythmia were 1.54 (95% CI, 1.02–2.33) and 2.65 (95% CI, 1.84–3.82) times more likely to develop embolic stroke, whereas current smoking, non-HDLC 150–169 and ≥170 mg/dL, HDLC <40 mg/dL and BMI ≥25 kg/m^2^ were 2.50 (95% CI, 1.58–3.96), 2.53 (95% CI, 1.34–4.76), 2.47 (95% CI, 1.34–4.53), 1.82 (95% CI, 1.15–2.87), and 1.58 (95% CI, 1.08–2.31) times more likely to develop large-artery occlusive stroke, respectively. The associations were generally similar between men and women (eTable 3A and eTable 3B).

**Table 3.** Multivariable-adjusted hazard ratios and population attributable fractions for ischemic stroke subtypes, JPHC Study, 1993–2012

Risk factors	Lacunar stroke		Embolic stroke		Large-artery occlusive stroke	
	*n* of cases = 233		*n* of cases = 179		*n* of cases = 121	

	HR (95% CI)	PAF (95% CI)	HR (95% CI)	PAF (95% CI)	HR (95% CI)	PAF (95% CI)
Men vs women	1.62 (1.19–2.20)		1.69 (1.21–2.38)		1.68 (1.09–2.58)	
Age per 1 year	1.10 (1.07–1.12)		1.11 (1.08–1.14)		1.11 (1.08–1.15)	
Current smoking vs non-smoking	1.35 (0.95–1.93)	6.1 (−1.6 to 13.1)	1.09 (0.71–1.66)	1.5 (−6.7 to 9.0)	2.50 (1.58–3.96)	18.8 (7.9–28.5)
Hypertension	1.77 (1.34–2.34)	28.2 (14.2–39.9)	1.76 (1.27–2.42)	28.4 (11.7–41.9)	1.39 (0.95–2.03)	16.9 (−4.2 to 33.7)
Diabetes mellitus	2.68 (1.84–3.91)	8.9 (4.0–13.5)	2.17 (1.36–3.47)	6.3 (1.2–11.2)	2.00 (1.11–3.61)	5.4 (−0.7 to 11.1)
Non-HDLC <130 vs 130–149 mg/dL	0.88 (0.61–1.26)	−4.1 (−16.0 to 6.7)	1.25 (0.82–1.91)	6.9 (−6.5 to 18.6)	1.79 (0.95–3.36)	12.0 (−1.0 to 23.4)
Non-HDLC 150–169 vs 130–149 mg/dL	0.76 (0.50–1.15)	−5.0 (−12.6 to 2.2)	0.98 (0.60–1.60)	−0.3 (−8.9 to 7.6)	2.53 (1.34–4.76)	15.5 (4.8–25.0)
Non-HDLC ≥170 vs 130–149 mg/dL	1.04 (0.73–1.49)	1.3 (−10.4 to 11.7)	1.18 (0.76–1.82)	4.5 (−7.9 to 15.5)	2.47 (1.34–4.53)	21.1 (7.5–32.7)
HDLC <40 vs ≥40 mg/dL	0.94 (0.62–1.43)	−0.7 (−5.5 to 3.9)	1.54 (1.02–2.33)	5.7 (−0.7 to 11.7)	1.82 (1.15–2.87)	9.3 (0.5–17.3)
Urine protein (+)	1.12 (0.71–1.74)	1.0 (−3.4 to 5.3)	1.33 (0.82–2.16)	2.8 (−2.5 to 7.8)	1.47 (0.85–2.54)	4.2 (−2.8 to 10.8)
Arrhythmia	0.97 (0.62–1.52)	−0.3 (−4.4 to 3.7)	2.65 (1.84–3.82)	13.2 (6.3–19.6)	1.02 (0.53–1.96)	0.1 (−5.4 to 5.4)
BMI <18.5 vs 18.5–<25 kg/m^2^	0.74 (0.33–1.69)	−0.9 (−3.1 to 1.2)	1.44 (0.69–2.98)	1.4 (−1.9 to 4.5)	0.31 (0.04–2.27)	−1.8 (−3.6 to −0.1)
BMI ≥25 vs 18.5–<25 kg/m^2^	1.12 (0.84–1.49)	3.8 (−6.2 to 12.8)	1.22 (0.88–1.68)	6.7 (−5.1 to 17.2)	1.58 (1.08–2.31)	17.2 (1.4–30.5)

BMI, body mass index; CI, confidence interval; HDLC, high-density lipoprotein cholesterol; HR, hazard ratio; JPHC Study, Japan Public Health Center-based Prospective Study; PAF, population attributable fraction; vs, versus.

Cox proportional hazard model that was mutually adjusted for all risk factors was used.

Hypertension was defined as systolic blood pressure ≥140 mm Hg, diastolic blood pressure ≥90 mm Hg, or the use of antihypertensive medication. Diabetes mellitus was defined as fasting blood glucose ≥126 mg/dL, casual blood glucose ≥200 mg/dL or use of antidiabetic medication. The non-HDLC ≥170 mg/dL category includes subjects who reported the use of lipid-lowering medication.

The associations between high normal and elevated blood pressure categories and total stroke were 1.26 (95% CI, 0.94–1.70) and 1.55 (95% CI, 1.21–1.98) compared to normal blood pressure, respectively (eTable 4 and eTable 5). The elevated blood pressure category was significantly associated with total, ischemic, and lacunar stroke incidence. The PAFs of high normal and elevated BP as well as of hypertension (Grade I, II, and III) in reference to normal BP for total stroke were 2.1%, 9.2%, and 26.7% (15.8 + 7.5 + 3.4), respectively (38.0% in total).

Hypertension accounted for about 30% of lacunar and embolic strokes, whereas diabetes mellites explained 8.9% and 6.3% of these subtypes, respectively. The PAF of arrhythmia was 13.2%, which was the second largest following hypertension for embolic stroke. Non-HDLC ≥150 (ie, 150–160 and ≥170) mg/dL, current smoking, BMI ≥25 kg/m^2^, and HDLC <40 mg/dL explained 36.6%, 18.8%, 17.2%, and 9.3% of large-artery occlusive strokes, respectively. Combined PAFs of all the modifiable risk factors for total, ischemic and intracerebral hemorrhage as well as each subtype of ischemic stroke (lacunar, embolic, and large-artery occlusive strokes) were 36.7%, 44.5%, 27.9%, 34.6%, 41.8%, and 61.5%, respectively. The PAFs2019 of hypertension for each ischemic stroke subtype were 3 to 4 percentage points lower. In contrast, those of diabetes mellitus were 6 to 8 percentage points higher than PAFs estimated in the present study.

## DISCUSSION

In the present study, consistent with previous reports,^19^ men had significantly higher risks of all types of strokes than women except for subarachnoid hemorrhage. Older age was consistently associated with all types of strokes. As to the magnitude of risk factors, hypertension alone accounted for more than a quarter of stroke incidence, followed by current smoking and diabetes. However, there are variations in the PAFs according to subtypes of stroke. High non-HDLC, current smoking, overweight, and low HDLC contributed most to large-artery occlusive stroke. Arrhythmia explained 13.2% of embolic stroke. Regarding the absolute values of the PAF due to hypertension, previous analysis of ours on the same JPHC study reported much higher values: 45.7% in men and 31.4% in women,^20^ as opposed to 26.2% in men and 24.9% in women in the present study. The exact reasons are not clear but would be related to longer follow-up in the present study (median follow-up, 20 vs 11 years). Also, we only studied Cohort II because of the availability of study variables of which age-range was 40–69 years compared to both Cohort I (40–59 years) and Cohort II in the previous study. In the analysis restricting age-range at baseline to 40–59 years in the Cohort II and the follow-up years to 10-year actually yielded similar estimates (data not shown).

Findings of the present study are consistent with previous literature. For example, the Hisayama Study found that non-HDLC was significantly positively associated with atherothrombotic stroke.^21^ However, the present study did not find an association between high non-HDLC and embolic infarction, although the Hisayama Study reported a significant inverse association. Similarly, low HDLC was related to large-artery occlusive and embolic strokes in the present study, whereas it was related to lacunar stroke and intracerebral hemorrhage in the Circulatory Risk in Communities Study (CIRCS)^22^ and Jichi Medical School Cohort Study.^23^ The strong association of blood pressure with stroke incidence is consistent with the CIRCS, in which high blood pressure, even within the normal range, was associated with higher stroke incidence.^24^ The highest PAF due to hypertension is also consistent with the previous reports.^4^

Although chronic kidney disease assessed as urine protein was associated with risks of both intracerebral hemorrhage and ischemc stroke, it did not seem to be associated with risk of ischemic stroke in men in the present study, which is consistent with the CIRCS.^25^ Also, the finding that BMI’s association was mostly explained by possible mediators with stroke incidence is consistent with a report of a pooled analysis of prospective studies.^26^ The elevated risks of subarachnoid hemorrhage associated with hypertenson in men and with current smoking in women would be consistent with previous reports from Japan.^27^^,^^28^ In contrast, we found significantly decreased risk of subarachnoid hemorrhge in relation to higher non-HDLC in women, which had not been observed previously.^29^ Furthermore, we did not find significant association between non-HDLC levels and risk of intracerebral hemorrhage, although previous studies reported an elevated risk of intracerebral hemorrhage in relation to low blood levels of low-density lipoprotein cholesterol.^2^^,^^30^^,^^31^ Especially, a seemingly different finding from our previous report from the JPHC Study was due to the different reference non-HDLC categories (130–149 in the present study vs <111 mg/dL in the previous report).

There are several limitations to the present study. First, innate to the long-term cohort study, the baseline survey was conducted approximately 20 years ago. Although the prevalence of hypertension remains high, pharmacological control of high blood pressure and clinical decision-making has improved since then.^6^ Also, the prevalence of smoking declined significantly in men in Japan in the past few decades. Since there are signs of an increase in other tobacco products, such as not-burn heated tobacco, and its health effects remain largely undetermined, relative and absolute risks related to smoking and tobacco products should be continuously monitored. We also calculated PAFs2019 using the current prevalence of risk factors. We found that PAFs and PAFs2019 were generally similar except for diabetes, of which contributions to total and ischemic strokes doubled. Second, the present study used a part of total cohort of which health check-up data and self-reported history of arrthymia was available. Those who were included in the study were older (57.6 vs 52.3 years) and were less likely to be current smoker (8% vs 3% in women and 55% vs 41% in men). The association as well as PAF estimates could be different if the entire cohort could have been used. Third, we used self-reported history of arrhythmia as a surrogate of atrial fibrillation. It could include other arrhythmic disorders than atrial fibrillation. Also, there are possibilities that those with atrial fibriallation did not report the history. Therefore, interpretation of the present results requires caution, but the fact that such a self-reported history was significantly associated with embolic stroke indicates the importance of atrial fibrillation as the risk factor. Fourth, blood pressure measurement using mercury sphygmomanometers is not recommended any more.^13^ Interpretation of the risks related to elevated blood pressure requires caution and further studies using currently recommended measurement methods should be carried out, since there may be systematic differences in the blood pressure readings.

There are several clinical and public health implications of the present study. First of all, the PAF as well as PAF2019 suggested the vital importance of prevention and control of hypertension for the prevention of stroke in Japan. Promoting primordial prevention of high blood pressure by adhering to healthy lifestyle throughout the life course is essential. This point could especially be emphasized as the PAF estimated using JSH 2019 blood pressure classification indicated more than a third of total stroke could be eliminated by preventing elevated blood pressure possibly through population-wide reduction of salt intake. Also, all Japanese aged 40 to 74 years are required to take annual health screening provided by their health insurer by law in Japan. Although awareness, treatment, and control of hypertension are reportedly improving,^32^ more efforts should be paid to encourage individuals to receive appropriate medical treatment utilizing the merits of universal health coverage in Japan. Furthermore, there are reports that clinicians are not always treating their patients to the level which the best available evidence-based methods would ideally expect. Strengthening communication between health insurers that provide health check-ups and clinicians in order for clinicians to increase and improve their involvement in prevention activities and avoid clinical inertia would be necessary. Second, the PAF2019 indicated that about 10% and 7% of stroke cases could be prevented by the elimination of diabetes and smoking, respectively. Since the prevalence of diabetes in Japan is not decreasing^17^^,^^33^^,^^34^ even with the national measures for the prevention, Tokutei Kenshin and Tokutei Hoken-Shidou (health check-up and control targeting metabolic syndrome). Populational prevention approaches, such as building healthy diet and a physical activity-promoting environment, is warranted. Regarding smoking, there has been a significant decline in the prevalence of cigarette smoking in Japan.^17^ Nevertheless, promoting smoking cessation and preventing tobacco products use by children and adolescents will remain an important public health issue. Third, ischemic stroke subtype analyses revealed that the highest PAF for large-artery occlusive stroke was elevated non-HDLC. Even though that for ischemic stroke was found to be not as large due to smaller composition of this type in the ischemic stroke, future increase in the population level of non-HDLC may change its public health importance for the prevention of stroke. Similarly, the PAF of self-reported arrhythmia, a surrogate of atrial fibrillation, was the second largest for embolic stroke. It is likely that atrial fibrillation, if more appropriately assessed, would show a higher PAF for embolic stroke. Prevention of atrial fibrillation and/or its appropriate treatment is of public health importance.

In conclusion, the present study described risk factors and their population impact for total and each subtype of stroke in Japan. Although there are differences in the subtypes, hypertension could be regarded as the most critical target for preventing strokes in Japan.

## SUPPLEMENTARY MATERIAL

eTable 1A. Multivariable-adjusted hazard ratios and population attributable fractions for stroke subtypes in men, JPHC Study, 1993–2012

eTable 1B. Multivariable-adjusted hazard ratios and population attributable fractions for stroke subtypes in women, JPHC Study, 1993–2012

eTable 2. Population attributable fraction estimated using multivariable-adjusted hazard ratios and the 2019 prevalence of risk factors for stroke and ischemic stroke subtypes (PAF2019), JPHC Study, 1993–2012

eTable 3A. Multivariable-adjusted hazard ratios and population attributable fractions for ischemic stroke subtypes in men, JPHC Study, 1993–2012

eTable 3B. Multivariable-adjusted hazard ratios and population attributable fractions for ischemic stroke subtypes in women, JPHC Study, 1993–2012

eTable 4. Multivariable-adjusted hazard ratios and population attributable fractions for stroke subtypes using JSH 2019 blood pressure classification, JPHC Study, 1993–2012

eTable 5. Multivariable-adjusted hazard ratios and population attributable fractions for ischemic stroke subtypes using JSH 2019 blood pressure classification, JPHC Study, 1993–2012
