# Supplementary material for: Risk and Population Attributable Fraction of Stroke Subtypes in Japan
Source: J Epidemiol. 2024 May 5;34(5):211–7. doi: 10.2188/jea.JE20220364 (PMC10999523; doi:10.2188/jea.JE20220364)
Supplement: Supplementary file 1 [file je-34-211-s001.pdf]

Supplementary material to: Yatsuya H, et al., Risk and population attributable fraction of stroke subtypes in Japan

eTable 1A. Multivariable-adjusted hazard ratios and population attributable fractions for stroke subtypes in men, JPHC Study, 1993−2012

	Total Stroke n of cases=447		Ischemic stroke n of cases=287		Intracerebral hemorrhage n of cases=134		Subarachnoid hemorrhage n of cases=12	
Risk factors	HR (95% CI)	PAF (95% CI)	HR (95% CI)	PAF (95% CI)	HR (95% CI)	PAF (95% CI)	HR (95% CI)	PAF (95% CI)
Age 40−69 years (per 1 year)	1.08 (1.06–1.09)		1.10 (1.08–1.12)		1.04 (1.01–1.07)		1.01 (0.93–1.10)	
Current smoking vs. non-smoking	1.29 (1.06–1.57)	9.3 (1.8–16.1)	1.54 (1.21–1.96)	15.9 (6.3–24.5)	0.80 (0.54–1.18)	-7.3 (-20.2 to 4.2)	1.74 (0.53–5.68)	21.2 (-40.8 to 55.9)
Hypertension (+)	1.70 (1.39–2.07)	26.2 (16.3–34.9)	1.63 (1.28–2.09)	24.6 (11.9–35.4)	1.80 (1.25–2.59)	28.2 (9.8–42.8)	5.97 (1.24–28.66)	69.4 (-10.0 to 91.5)
Diabetes mellitus (+)	1.80 (1.34–2.42)	5.2 (1.9–8.3)	1.94 (1.35–2.77)	6.1 (1.8–10.1)	1.27 (0.68–2.39)	1.8 (-3.4 to 6.7)	2.60 (0.55–12.27)	10.3 (-15.7 to 30.4)
Non-HDLC <130 vs. 130−149 mg/dL	1.11 (0.87–1.42)	4.1 (-5.8 to 13.1)	1.03 (0.76–1.41)	1.3 (-11.4 to 12.5)	1.27 (0.81–1.98)	9.3 (-9.2 to 24.7)	1.11 (0.27–4.55)	5.0 (-89.9 to 52.4)
Non-HDLC 150−169 vs. 130−149 mg/dL	0.94 (0.69–1.28)	-1.0 (-5.9 to 3.7)	0.85 (0.57–1.27)	-2.3 (-8.3 to 3.3)	1.22 (0.72–2.06)	3.6 (-6.5 to 12.8)	NA	NA
Non-HDLC ≥170 vs. 130−149 mg/dL	1.03 (0.78–1.37)	0.7 (-5.4 to 6.5)	1.24 (0.88–1.74)	4.7 (-3.2 to 12.1)	0.63 (0.35–1.14)	-7.9 (-18.2 to 1.5)	1.15 (0.23–5.77)	3.2 (-43.1 to 34.5)
HDLC <40 vs. ≥40 mg/dL	1.19 (0.92–1.55)	2.5 (-1.6 to 6.5)	1.08 (0.77–1.50)	1.1 (-4.1 to 6.0)	1.46 (0.91–2.34)	5.1 (-2.4 to 12.1)	2.31 (0.59–8.97)	14.2 (-19.4 to 38.3)
Urine protein (+)	1.22 (0.91–1.65)	2.2 (-1.3 to 5.6)	1.18 (0.81–1.71)	1.8 (-2.6 to 6.0)	1.47 (0.88–2.45)	4.3 (-2.5 to 10.6)	0.94 (0.11–7.75)	-0.5 (-19.9 to 15.7)
Arrhythmia (+)	1.36 (1.01–1.82)	3.1 (-0.3 to 6.3)	1.42 (1.00–2.03)	3.7 (-0.6 to 7.9)	1.22 (0.68–2.17)	1.7 (-3.9 to 7.1)	2.06 (0.44–9.71)	8.6 (-17.9 to 29.1)
BMI <18.5 vs. 18.5−<25 kg/m^2	0.73 (0.40–1.35)	-0.9 (-2.4 to 0.6)	0.67 (0.32–1.44)	-1.2 (-3.1 to 0.7)	0.81 (0.25–2.59)	-0.5 (-3.2 to 2.1)	3.06 (0.36–26.33)	5.6 (-12.1 to 20.5)
BMI ≥25 vs. 18.5−<25 kg/m^2	1.17 (0.94–1.44)	4.8 (-2.1 to 11.3)	1.12 (0.86–1.47)	3.5 (-4.9 to 11.2)	1.13 (0.77–1.66)	4.3 (-10.0 to 16.7)	0.65 (0.16–2.71)	-13.3 (-65.4 to 22.4)

BMI, body mass index; CI, confidence interval; HDLC, high-density lipoprotein cholesterol; HR, hazard ratio; JPHC Study, Japan Public Health Center-based Prospective Study; PAF, population attributable fraction; vs., versus.

Cox proportional hazard model that was mutually adjusted for all risk factors was used.

Hypertension was defined as systolic blood pressure ≥140 mm Hg, diastolic blood pressure ≥90 mm Hg, or self-reported use of antihypertensive medication. Diabetes mellitus was defined as fasting blood glucose ≥126 mg/dL, casual blood glucose ≥200 mg/dL or the use of antidiabetic medication. The non-HDLC ≥170 mg/dL category includes subjects who reported the use of lipid-lowering medication.

eTable 1B. Multivariable-adjusted hazard ratios and population attributable fractions for stroke subtypes in women, JPHC Study, 1993−2012

	Total Stroke		Ischemic stroke		Intracerebral hemorrhage		Subarachnoid hemorrhage	
	n of cases=468		n of cases=285		n of cases=117		n of cases=58	
Risk factors	HR (95% CI)	PAF (95% CI)	HR (95% CI)	PAF (95% CI)	HR (95% CI)	PAF (95% CI)	HR (95% CI)	PAF (95% CI)
Age 40−69 years (per 1 year)	1.10 (1.08–1.12)		1.12 (1.09–1.14)		1.09 (1.06–1.12)		1.06 (1.02–1.10)	
Current smoking vs. non-smoking	1.70 (1.03–2.81)	1.4 (-0.3 to 3.1)	1.69 (0.90–3.19)	1.4 (-0.8 to 3.6)	1.01 (0.25–4.13)	0.0 (-2.4 to 2.4)	3.03 (1.08–8.46)	4.6 (-2.3 to 11.1)
Hypertension (+)	1.67 (1.37–2.03)	24.9 (15.2–33.4)	1.57 (1.22–2.03)	23.2 (9.9–34.6)	1.85 (1.25–2.73)	27.1 (8.8–41.7)	1.44 (0.83–2.51)	1 6.9 (-12.0 to 38.4)
Diabetes mellitus (+)	1.87 (1.33–2.62)	3.8 (1.1–6.3)	2.74 (1.89–3.96)	7.3 (3.4–11.2)	0.57 (0.18–1.81)	-1.9 (-5.1 to 1.1)	0.41 (0.06–2.98)	-2.5 (-6.1 to 1.0)
Non-HDLC <130 vs. 130−149 mg/dL	1.26 (0.96–1.65)	5.3 (-1.2 to 11.4)	1.30 (0.89–1.89)	5.2 (-2.6 to 12.4)	1.41 (0.83–2.40)	9.0 (-5.4 to 21.5)	0.92 (0.48–1.75)	-2.9 (-27.5 to 17.0)
Non-HDLC 150−169 vs. 130−149 mg/dL	0.97 (0.73–1.30)	-0.5 (-6.4 to 5.0)	1.28 (0.88–1.87)	4.9 (-2.7 to 11.9)	0.78 (0.42–1.45)	-4.3 (-15.6 to 5.9)	0.37 (0.16–0.88)	-20.5 (-38.0 to -5.2)
Non-HDLC ≥170 vs. 130−149 mg/dL	1.12 (0.87–1.45)	3.8 (-5.1 to 11.9)	1.34 (0.95–1.89)	9.7 (-1.8 to 19.9)	1.19 (0.71–1.99)	5.4 (-11.9 to 20.1)	0.44 (0.21–0.89)	-29.1 (-57.5 to -5.8)
HDLC <40 vs. ≥40 mg/dL	1.39 (1.04–1.87)	3.1 (-0.1 to 6.2)	1.64 (1.16–2.31)	5.3 (0.8–9.6)	0.86 (0.40–1.87)	-1.0 (-5.8 to 3.6)	1.09 (0.43–2.76)	0.7 (-7.6 to 8.3)
Urine protein (+)	1.43 (1.05–1.94)	3.1 (0.0–6.2)	1.42 (0.96–2.10)	3.1 (-0.9 to 6.9)	1.55 (0.85–2.80)	3.9 (-2.5 to 10.0)	1.40 (0.55–3.61)	2.5 (-5.7 to 10.0)
Arrhythmia (+)	1.30 (0.97–1.75)	2.5 (-0.6 to 5.6)	1.59 (1.12–2.24)	4.9 (0.5–9.2)	0.64 (0.28–1.46)	-2.9 (-7.4 to 1.4)	1.23 (0.53–2.89)	1.9 (-7.1 to 10.2)
BMI <18.5 vs. 18.5−<25 kg/m^2	0.92 (0.54–1.59)	-0.2 (-1.9 to 1.4)	0.95 (0.47–1.95)	-0.1 (-2.2 to 1.9)	0.91 (0.33–2.50)	-0.4 (-3.9 to 3.1)	0.47 (0.06–3.44)	-2.0 (-5.7 to 1.6)
BMI ≥25 vs. 18.5−<25 kg/m^2	1.13 (0.93–1.37)	4.6 (-3.1 to 11.7)	1.32 (1.03–1.68)	10.5 (0.4–19.6)	0.67 (0.44–1.01)	-14.6 (-29.9 to -1.2)	1.62 (0.94–2.78)	17.1 (-5.2 to 34.7)

BMI, body mass index; CI, confidence interval; HDLC, high-density lipoprotein cholesterol; HR, hazard ratio; JPHC Study, Japan Public Health Center-based Prospective Study; PAF, population attributable fraction; vs., versus.

Cox proportional hazard model that was mutually adjusted for all the risk factors was used.

Hypertension was defined as systolic blood pressure ≥140 mm Hg, diastolic blood pressure ≥90 mm Hg, or the use of antihypertensive medication. Diabetes mellitus was defined as fasting blood glucose ≥126 mg/dL, casual blood glucose ≥200 mg/dL or the use of antidiabetic medication. The non-HDLC ≥170 mg/dL category includes subjects who reported the use of lipid-lowering medication.

eTable 2. Population attributable fraction estimated using multivariable-adjusted hazard ratios and the 2019 prevalence of risk factors for stroke and ischemic stroke subtypes (PAF2019), JPHC Study, 1993−2012

Risk factors	Total Stroke	Ischemic stroke	Intracerebral hemorrhage	Subarachnoid hemorrhage	Lacunar stroke	Embolic stroke	Large-artery occlusive stroke
Current smoking vs. non-smoking	6.6	10.5	-3.7	21.7	7.0	1.8	24.0
Hypertension (+)	22.5	20.8	25.9	23.8	24.4	24.0	14.0
Diabetes mellitus (+)	8.9	12.7	0.3	-1.2	16.4	12.1	10.5
Non-HDLC <130 vs. 130−149 mg/dL	3.4	2.9	6.0	-1.2	-2.7	5.1	14.5
Non-HDLC 150−169 vs. 130−149 mg/dL	-0.9	1.1	0.2	-14.8	-5.0	-0.3	22.9
Non-HDLC ≥170 vs. 130−149 mg/dL	2.4	7.7	-2.2	-18.5	1.3	5.2	31.0
HDLC <40 vs. ≥40 mg/dL	0.9	0.9	0.9	1.0	-0.2	1.8	2.6
Urine protein (+)	1.2	1.1	1.8	1.1	0.4	1.3	1.8
Arrhythmia (+)	1.1	1.6	-0.1	1.2	-0.1	5.0	0.1
BMI <18.5 vs. 18.5−<25 kg/m^2	-1.2	-1.5	-0.9	-1.3	-1.9	3.1	-5.2
BMI ≥25 vs. 18.5−<25 kg/m^2	4.4	6.0	-3.2	11.4	3.4	6.0	14.5

BMI, body mass index; HDLC, high-density lipoprotein cholesterol; JPHC Study, Japan Public Health Center-based Prospective Study; PAF, population attributable fraction; vs., versus.

JPHC Study indicates Japan Public Health Center-based Prospective Study; HR, hazard ratio; PAF, population attributable fraction; vs., versus; HDL, high-density lipoprotein; BMI, body mass index.

Hypertension was defined as systolic blood pressure ≥140 mm Hg, diastolic blood pressure ≥90 mm Hg, or the use of antihypertensive medication. Diabetes mellitus was defined as fasting blood glucose ≥126 mg/dL, casual blood glucose ≥200 mg/dL or the use of antidiabetic medication. The non-HDLC ≥170 mg/dL category includes subjects who reported the use of lipid-lowering medication.

eTable 3A. Multivariable-adjusted hazard ratios and population attributable fractions for ischemic stroke subtypes in men, JPHC Study, 1993–2012

	Lacunar stroke n of cases=114		Embolic stroke n of cases=86		Large-artery occlusive stroke n of cases=66	
Risk factors	HR (95% CI)	PAF (95% CI)	HR (95% CI)	PAF (95% CI)	HR (95% CI)	PAF (95% CI)
Age 40–69 years (per 1 year)	1.09 (1.05–1.12)		1.10 (1.06–1.15)		1.11 (1.07–1.16)	
Current smoking vs. non-smoking	1.41 (0.97–2.07)	13.1 (-2.9 to 26.6)	1.16 (0.74–1.83)	5.4 (-12.4 to 20.4)	2.26 (1.37–3.73)	28.7 (8.3–44.6)
Hypertension (+)	1.79 (1.20–2.65)	28.6 (7.9–44.6)	2.17 (1.35–3.49)	37.7 (13.6–55.0)	1.26 (0.76–2.09)	12.0 (-17.5 to 34.0)
Diabetes mellitus (+)	2.22 (1.29–3.81)	7.7 (0.6–14.3)	1.63 (0.83–3.21)	4.5 (-3.2 to 11.6)	2.23 (1.08–4.60)	7.5 (-1.9 to 16.0)
Non-HDLC <130 vs. 130–149 mg/dL	0.83 (0.52–1.33)	-7.8 (-30.1 to 10.7)	0.97 (0.57–1.65)	-1.5 (-27.2 to 19.1)	1.88 (0.80–4.46)	14.9 (-5.4 to 31.3)
Non-HDLC 150–169 vs. 130–149 mg/dL	0.52 (0.26–1.03)	-9.1 (-18.1 to -0.7)	0.63 (0.30–1.34)	-6.8 (-17.8 to 3.2)	3.45 (1.44–8.28)	19.4 (5.2–31.5)
Non-HDLC ≥170 vs. 130–149 mg/dL	1.07 (0.64–1.80)	1.7 (-11.9 to 13.6)	0.90 (0.48–1.69)	-2.4 (-17.3 to 10.6)	3.05 (1.28–7.27)	20.4 (4.6–33.6)
HDLC <40 vs. ≥40 mg/dL	0.78 (0.43–1.41)	-3.3 (-10.6 to 3.6)	1.36 (0.76–2.43)	4.6 (-5.3 to 13.7)	1.41 (0.76–2.61)	6.2 (-6.7 to 17.4)
Urine protein (+)	1.10 (0.60–2.01)	1.0 (-6.0 to 7.6)	1.30 (0.67–2.51)	2.9 (-5.5 to 10.7)	1.16 (0.53–2.51)	1.6 (-7.9 to 10.4)
Arrhythmia (+)	0.71 (0.35–1.47)	-2.8 (-8.3 to 2.3)	2.59 (1.52–4.42)	12.9 (2.8–21.9)	1.56 (0.74–3.29)	4.3 (-4.7 to 12.6)
BMI <18.5 vs. 18.5–<25 kg/m^2	0.69 (0.22–2.21)	-1.2 (-4.4 to 1.9)	1.08 (0.33–3.51)	0.3 (-3.9 to 4.3)	0.54 (0.07–3.96)	-36.7 (-327.5 to 56.3)
BMI ≥25 vs. 18.5–<25 kg/m^2	1.02 (0.66–1.58)	0.6 (-12.4 to 12.1)	1.03 (0.63–1.70)	1.0 (-15.0 to 14.8)	1.55 (0.92–2.64)	35.6 (-15.8 to 64.2)

BMI, body mass index; CI, confidence interval; HDLC, high-density lipoprotein cholesterol; HR, hazard ratio; JPHC Study, Japan Public Health Center-based Prospective Study; PAF, population attributable fraction; vs., versus.

Cox proportional hazard model that was mutually adjusted for all the risk factors was used.

Hypertension was defined as systolic blood pressure ≥140 mm Hg, diastolic blood pressure ≥90 mm Hg, or the use of antihypertensive medication. Diabetes mellitus was defined as fasting blood glucose ≥126 mg/dL, casual blood glucose ≥200 mg/dL or the use of antidiabetic medication. The non-HDLC ≥170 mg/dL category includes subjects who reported the use of lipid-lowering medication.

eTable 3B. Multivariable-adjusted hazard ratios and population attributable fractions for ischemic stroke subtypes in women, JPHC Study, 1993–2012

	Lacunar stroke n of cases=119		Embolic stroke n of cases=93		Large-artery occlusive stroke n of cases=55	
Risk factors	HR (95% CI)	PAF (95% CI)	HR (95% CI)	PAF (95% CI)	HR (95% CI)	PAF (95% CI)
Age 40−69 years (per 1 year)	1.10 (1.07–1.14)		1.11 (1.07–1.15)		1.11 (1.06–1.17)	
Current smoking vs. non-smoking	1.08 (0.34–3.42)	0.2 (-2.7 to 3.0)	0.52 (0.07–3.72)	-1.0 (-3.2 to 1.1)	4.07 (1.45–11.46)	5.5 (-1.8 to 12.2)
Hypertension (+)	1.69 (1.13–2.50)	26.3 (5.2–42.7)	1.41 (0.90–2.19)	18.0 (-7.7 to 37.5)	1.56 (0.87–2.79)	22.9 (-10.9 to 46.4)
Diabetes mellitus (+)	3.50 (2.07–5.91)	10.2 (3.4–16.6)	2.94 (1.54–5.61)	7.8 (0.7–14.4)	1.53 (0.54–4.32)	2.5 (-5.0 to 9.5)
Non-HDLC <130 vs. 130−149 mg/dL	0.93 (0.53–1.63)	-1.5 (-14.0 to 9.6)	1.75 (0.89–3.43)	11.5 (-2.9 to 23.9)	1.78 (0.70–4.56)	9.6 (-7.0 to 23.5)
Non-HDLC 150−169 vs. 130−149 mg/dL	0.98 (0.57–1.72)	-0.3 (-12.8 to 10.8)	1.54 (0.76–3.09)	7.5 (-5.3 to 18.8)	1.72 (0.68–4.32)	9.9 (-7.9 to 24.7)
Non-HDLC ≥170 vs. 130−149 mg/dL	1.05 (0.64–1.71)	1.7 (-18.3 to 18.2)	1.59 (0.84–3.03)	14.0 (-5.8 to 30.1)	1.94 (0.83–4.54)	20.2 (-6.3 to 40.1)
HDLC <40 vs. ≥40 mg/dL	1.25 (0.70–2.23)	2.2 (-4.3 to 8.2)	1.92 (1.07–3.44)	7.2 (-1.1 to 14.9)	2.54 (1.29–5.02)	12.1 (-0.4 to 23.1)
Urine protein (+)	1.13 (0.58–2.21)	1.0 (-4.7 to 6.3)	1.38 (0.68–2.80)	2.6 (-4.2 to 9.0)	1.89 (0.87–4.12)	6.9 (-4.0 to 16.6)
Arrhythmia (+)	1.26 (0.71–2.25)	2.2 (-4.1 to 8.2)	2.73 (1.65–4.52)	13.6 (3.9–22.4)	0.41 (0.10–1.70)	-5.2 (-10.8 to 0.2)
BMI <18.5 vs. 18.5–25 kg/m^2	0.80 (0.25–2.55)	-0.6 (-3.7 to 2.3)	1.94 (0.76–4.91)	2.6 (-2.3 to 7.2)	NA	NA
BMI ≥25 vs. 18.5–<25 kg/m^2	1.23 (0.84–1.80)	7.7 (-7.8 to 21.0)	1.44 (0.93–2.22)	13.4 (-4.5 to 28.3)	1.55 (0.89–2.71)	18.7 (-8.5 to 39.1)

BMI, body mass index; CI, confidence interval; HDLC, high-density lipoprotein cholesterol; HR, hazard ratio; JPHC Study, Japan Public Health Center-based Prospective Study; PAF, population attributable fraction; vs., versus.

Cox proportional hazard model that was mutually adjusted for all risk factors was used.

Hypertension was defined as systolic blood pressure ≥140 mm Hg, diastolic blood pressure ≥90 mm Hg, or the use of antihypertensive medication. Diabetes mellitus was defined as fasting blood glucose ≥126 mg/dL, casual blood glucose ≥200 mg/dL or the use of antidiabetic medication. The non-HDLC ≥170 mg/dL category includes subjects who reported the use of lipid-lowering medication.

eTable 4. Multivariable-adjusted hazard ratios and population attributable fractions for stroke subtypes using JSH 2019 blood pressure classification, JPHC Study, 1993−2012

	Total Stroke n of cases=915		Ischemic stroke n of cases=572		Intracerebral hemorrhage n of cases=251		Subarachnoid hemorrhage n of cases=70	
Risk factors	HR (95% CI)	PAF (95% CI)	HR (95% CI)	PAF (95% CI)	HR (95% CI)	PAF (95% CI)	HR (95% CI)	PAF (95% CI)
Men vs. women	1.58 (1.36–1.84)		1.64 (1.35–1.99)		2.02 (1.53–2.66)		0.26 (0.12–0.55)	
Age 40−69 years	1.09 (1.08–1.10)		1.11 (1.09–1.12)		1.06 (1.04–1.08)		1.05 (1.01–1.08)	
Current smoking vs. non-smoking	1.34 (1.12–1.61)	5.5 (1.9–9.0)	1.57 (1.25–1.96)	8.8 (4.1–13.4)	0.84 (0.58–1.22)	-3.0 (-9.6 to 3.1)	2.32 (1.03–5.25)	8.1 (-1.6 to 16.9)
High normal vs. Normal BP	1.26 (0.94–1.70)	2.1 (-0.6 to 4.7)	1.07 (0.73–1.57)	0.6 (-2.7 to 3.9)	1.36 (0.79–2.34)	2.8 (-2.4 to 7.8)	2.73 (1.02–7.31)	10.9 (-0.5 to 20.9)
Elevated BP vs. Normal BP	1.55 (1.21–1.98)	9.2 (4.2–13.9)	1.56 (1.15–2.13)	10.2 (3.5–16.4)	1.44 (0.90–2.29)	7.1 (-2.0 to 15.5)	1.88 (0.73–4.88)	10.7 (-5.3 to 24.3)
Grade I hypertensin vs. Normal BP	1.79 (1.40–2.29)	15.8 (9.8–21.4)	1.65 (1.21–2.24)	14.5 (6.3–22.1)	1.94 (1.23–3.08)	16.3 (5.9–25.5)	2.44 (0.95–6.22)	19.4 (0.4–34.7)
Grade II hypertensin vs. Normal BP	2.27 (1.71–3.03)	7.5 (4.8–10.1)	1.75 (1.21–2.54)	5.0 (1.6–8.2)	3.31 (1.98–5.52)	11.7 (6.2–16.8)	3.30 (1.12–9.75)	9.0 (-0.3 to 17.4)
Grade III hypertensin vs. Normal BP	3.05 (2.10–4.41)	3.4 (1.9–4.9)	2.47 (1.53–3.99)	2.8 (0.9–4.6)	3.84 (1.92–7.68)	3.8 (1.0–6.6)	5.59 (1.48–21.14)	4.7 (-1.0 to 10.1)
Diabetes mellitus (+)	2.17 (1.68–2.80)	5.3 (3.2–7.4)	2.67 (2.00–3.57)	7.5 (4.6–10.4)	1.15 (0.59–2.26)	0.7 (-2.9 to 4.2)	0.92 (0.22–3.78)	-0.4 (-6.9 to 5.7)
Non-HDLC <130 vs. 130−149 mg/dL	1.17 (0.98–1.41)	4.9 (-0.8 to 10.3)	1.15 (0.90–1.46)	4.0 (-3.1 to 10.6)	1.31 (0.93–1.84)	8.9 (-2.7 to 19.2)	0.95 (0.53–1.70)	-1.8 (-25.6 to 17.5)
Non-HDLC 150−169 vs. 130−149 mg/dL	0.96 (0.78–1.19)	-0.7 (-4.5 to 2.9)	1.06 (0.81–1.39)	1.0 (-3.8 to 5.6)	1.02 (0.69–1.52)	0.4 (-7.0 to 7.2)	0.34 (0.15–0.80)	-19.5 (-34.0 to -6.6)
Non-HDLC ≥170 vs. 130−149 mg/dL	1.08 (0.89–1.30)	2.0 (-3.3 to 7.0)	1.27 (1.00–1.61)	6.6 (-0.19 to 13.0)	0.93 (0.64–1.35)	-1.8 (-11.3 to 6.9)	0.49 (0.25–0.93)	-24.1 (-48.5 to -3.6)
HDLC <40 vs. ≥40 mg/dL	1.27 (1.05–1.55)	2.9 (0.3–5.4)	1.28 (1.01–1.63)	3.2 (-0.2 to 6.5)	1.27 (0.86–1.90)	2.5 (-2.1 to 6.8)	1.30 (0.61–2.76)	2.6 (-6.1 to 10.6)
Urine protein (+)	1.29 (1.04–1.59)	2.5 (0.2–4.8)	1.28 (0.98–1.68)	2.5 (-0.5 to 5.3)	1.39 (0.94–2.06)	3.5 (-1.2 to 7.9)	1.17 (0.49–2.79)	1.3 (-6.3 to 8.3)
Arrhythmia (+)	1.34 (1.09–1.65)	2.9 (0.6–5.1)	1.50 (1.17–1.92)	4.3 (1.2–7.3)	0.98 (0.61–1.58)	-0.1 (-3.8 to 3.4)	1.36 (0.65–2.86)	3.0 (-5.6 to 10.9)
BMI <18.5 vs. 18.5−<25 kg/m^2	0.86 (0.58–1.29)	-0.4 (-1.6 to 0.7)	0.83 (0.49–1.40)	-0.5 (-1.9 to 0.8)	0.89 (0.42–1.92)	-0.3 (-2.5 to 1.8)	0.89 (0.21–3.73)	-0.3 (-4.6 to 3.7)
BMI ≥25 vs. 18.5−<25 kg/m^2	1.13 (0.98–1.31)	4.3 (-0.9 to 9.2)	1.19 (0.99–1.43)	6.0 (-0.5 to 12.1)	0.88 (0.67–1.17)	-4.5 (-14.8 to 4.9)	1.38 (0.83–2.29)	11.5 (-8.7 to 27.9)

BMI, body mass index; BP, blood pressure; CI, confidence interval; HDLC, high-density lipoprotein cholesterol; HR, hazard ratio; JPHC Study, Japan Public Health Center-based Prospective Study; PAF, population attributable fraction; vs., versus.

Cox proportional hazard model that included all the risk factors as well as the interaction terms of blood pressure (BP) categories with antihypertensive medication use was used.

Normal BP was defined systolic BP (SBP) <120 and diastolic (DBP) <80; high normal BP, SBP 120–129 and DBP <80; elevated BP, SBP 130–139 and/or DBP 80–89 mm Hg; Grade I hypertension, SBP 140−159 and/or DBP 90−99 mm Hg; Grade II hypertension, SBP 160−179 and/or DBP 100−109 mm Hg, Grade III hypertension, SBP ≥180 and/or DBP ≥110 mm Hg. Diabetes mellitus was defined as fasting blood glucose ≥126 mg/dL, casual blood glucose ≥200 mg/dL or the use of antidiabetic medication. The non-HDLC ≥170 mg/dL category includes subjects who reported the use of lipid-lowering medication.

eTable 5. Multivariable-adjusted hazard ratios and population attributable fractions for ischemic stroke subtypes using JSH 2019 blood pressure classification, JPHC Study, 1993–2012

	Lacunar stroke n of cases=233		Embolic stroke n of cases=179		Large-artery occlusive stroke n of cases=121	
Risk factors	HR (95% CI)	PAF (95% CI)	HR (95% CI)	PAF (95% CI)	HR (95% CI)	PAF (95% CI)
Men vs. women	1.62 (1.19–2.20)		1.72 (1.22–2.42)		1.67 (1.08–2.57)	
Age 40–69 years	1.09 (1.07–1.12)		1.11 (1.08–1.14)		1.11 (1.08–1.15)	
Current smoking vs. non-smoking	1.34 (0.93–1.91)	5.8 (-1.9 to 12.9)	1.11 (0.73–1.70)	1.9 (-6.2 to 9.4)	2.50 (1.58–3.98)	18.9 (7.9–28.5)
High normal vs. Normal BP	1.19 (0.63–2.26)	1.3 (-3.6 to 6.0)	0.83 (0.42–1.67)	-1.6 (-7.7 to 4.2)	0.54 (0.22–1.33)	-4.9 (-12.0 to 1.8)
Elevated BP vs. Normal BP	2.07 (1.24–3.45)	16.0 (5.7–25.1)	1.20 (0.69–2.07)	4.0 (-8.5 to 14.9)	1.30 (0.71–2.40)	6.9 (-9.6 to 21.0)
Grade I hypertensin vs. Normal BP	2.15 (1.29–3.59)	20.7 (8.5–31.2)	1.39 (0.81–2.37)	10.3 (-6.5 to 24.4)	1.37 (0.75–2.51)	10.2 (-10.1 to 26.9)
Grade II hypertensin vs. Normal BP	2.00 (1.07–3.71)	5.1 (0.3–9.8)	1.73 (0.92–3.25)	5.7 (-1.1 to 11.9)	1.33 (0.62–2.87)	2.7 (-4.9 to 9.7)
Grade III hypertensin vs. Normal BP	2.64 (1.17–5.99)	2.4 (-0.2 to 5.0)	3.15 (1.50–6.62)	5.0 (0.8–8.9)	1.42 (0.46–4.43)	1.0 (-2.6 to 4.4)
Diabetes mellitus	3.52 (2.35–5.28)	10.1 (5.3–14.8)	2.32 (1.33–4.05)	6.7 (1.3–11.8)	2.05 (0.99–4.24)	5.5 (-1.0 to 11.6)
Non-HDLC <130 vs. 130–149 mg/dL	0.89 (0.62–1.27)	-3.8 (-15.7 to 6.9)	1.25 (0.82–1.90)	6.8 (-6.7 to 18.5)	1.80 (0.96–3.39)	12.1 (-0.8 to 23.4)
Non-HDLC 150–169 vs. 130–149 mg/dL	0.76 (0.50–1.16)	-4.9 (-12.6 to 2.2)	0.98 (0.60–1.60)	-0.3 (-9.0 to 7.6)	2.54 (1.35–4.79)	15.5 (4.8–25.1)
Non-HDLC ≥170 vs. 130–149 mg/dL	1.03 (0.72–1.47)	0.9 (-10.9 to 11.4)	1.17 (0.76–1.81)	4.4 (-8.1 to 15.4)	2.46 (1.34–4.53)	21.1 (7.5–32.7)
HDLC <40 vs. ≥40 mg/dL	0.95 (0.63–1.45)	-0.5 (-5.3 to 4.0)	1.52 (1.01–2.29)	5.5 (-0.9 to 11.5)	1.84 (1.17–2.92)	9.5 (0.7–17.4)
Urine protein (+)	1.14 (0.73–1.78)	1.2 (-3.2 to 5.4)	1.25 (0.77–2.03)	2.2 (-3.1 to 7.3)	1.52 (0.88–2.63)	4.5 (-2.5 to 11.1)
Arrhythmia (+)	0.97 (0.62–1.53)	-0.3 (-4.5 to 3.7)	2.62 (1.81–3.77)	13.1 (6.2–19.5)	1.04 (0.54–2.00)	0.3 (-5.2 to 5.6)
BMI <18.5 vs. 18.5–<25 kg/m^2	0.77 (0.34–1.74)	-0.8 (-3.0 to 1.3)	1.53 (0.74–3.17)	1.5 (-1.7 to 4.7)	0.32 (0.04–2.29)	-1.8 (-3.5 to -0.1)
BMI ≥25 vs. 18.5–<25 kg/m^2	1.10 (0.83–1.47)	3.2 (-6.8 to 12.4)	1.17 (0.85–1.62)	5.4 (-6.7 to 16.2)	1.55 (1.06–2.28)	16.7 (0.7–30.1)

BMI, body mass index; BP, blood pressure; CI, confidence interval; HDLC, high-density lipoprotein cholesterol; HR, hazard ratio; JPHC Study, Japan Public Health Center-based Prospective Study; PAF, population attributable fraction; vs., versus.

Cox proportional hazard model that included all the risk factors as well as the interaction terms of BP categories with antihypertensive medication use was used.

Normal BP was defined as systolic BP (SBP) <120 and diastolic BP (DBP) <80; high normal BP, SBP 120–129 and DBP <80; elevated BP, SBP 130–139 and/or DBP 80–89 mm Hg; Grade I hypertension, SBP 140–159 and/or DBP 90–99 mm Hg; Grade II hypertension, SBP 160–179 and/or DBP 100-109 mm Hg, Grade III hypertension, SBP ≥180 and/or DBP ≥110 mm Hg. Diabetes mellitus was defined as fasting blood glucose ≥126 mg/dL, casual blood glucose ≥200 mg/dL or the use of antidiabetic medication. The non-HDLC ≥170 mg/dL category includes subjects who reported the use of lipid-lowering medication.
